# A Three-Dimensional Mechanical Loading Model of Human Osteocytes in Their Native Matrix

**DOI:** 10.1007/s00223-021-00919-z

**Published:** 2021-10-13

**Authors:** Chen Zhang, Elisabet Farré-Guasch, Jianfeng Jin, Huib W. van Essen, Jenneke Klein-Nulend, Nathalie Bravenboer

**Affiliations:** 1 Department of Oral Cell Biology, Academic Centre for Dentistry Amsterdam (ACTA), University of Amsterdam and Vrije Universiteit Amsterdam, Amsterdam Movement Sciences, Amsterdam, The Netherlands; 2 Department of Clinical Chemistry, Amsterdam University Medical Centers, Vrije Universiteit Amsterdam, Amsterdam Movement Sciences, P.O. Box 7057, 1007 MB Amsterdam, The Netherlands

**Keywords:** Three-dimensional, Bone microdamage, Human cortical bone, Mechanical loading, Native matrix, Osteocytes

## Abstract

Osteocytes are mechanosensory cells which are embedded in calcified collagenous matrix. The specific native matrix of osteocytes affects their regulatory activity, i.e., transmission of signaling molecules to osteoclasts and/or osteoblasts, in the mechanical adaptation of bone. Unfortunately, no existing in vitro model of cortical bone is currently available to study the mechanosensory function of human osteocytes in their native matrix. Therefore, we aimed to develop an in vitro three-dimensional mechanical loading model of human osteocytes in their native matrix. Human cortical bone explants containing osteocytes in their three-dimensional native matrix were cultured and mechanically loaded by three-point bending using a custom-made loading apparatus generating sinusoidal displacement. Osteocyte viability and sclerostin expression were measured 1–2 days before 5 min loading and 1 day after loading. Bone microdamage was visualized and quantified by micro-CT analysis and histology using BaSO_4_ staining. A linear relationship was found between loading magnitude (2302–13,811 µɛ) and force (1.6–4.9 N) exerted on the bone explants. At 24 h post-loading, osteocyte viability was not affected by 1600 µɛ loading. Sclerostin expression and bone microdamage were unaffected by loading up to 8000 µɛ. In conclusion, we developed an in vitro 3D mechanical loading model to study mechanoresponsiveness of viable osteocytes residing in their native matrix. This model is suitable to study the effect of changed bone matrix composition in metabolic bone disease on osteocyte mechanoresponsiveness.

## Introduction

Osteocytes are the most abundant cells in bone, comprising 90–95% of all bone cells. However, their function is the least studied [1]. In bone, osteocytes are surrounded by a calcified collagenous matrix. They are connected with each other through their cell processes running through canaliculi that form an extensive canalicular network throughout the bone. As the principle mechanosensory bone cells, osteocytes play a pivotal role in the adaptation of bone to mechanical stimuli [2]. Mechanical loading causes deformation of bone, which creates a pressure gradient in the interstitial fluid present in the lacuno-canalicular network surrounding the osteocytes. This pressure gradient induces mechanical stimuli in the form of fluid shear stress [1]. Osteocytes sense this fluid shear stress by their processes and convert the mechanical stimuli into chemical signals such as nitric oxide (NO) and prostaglandin E_2_ [3]. Osteocytes orchestrate bone adaptation to mechanical loading by steering bone formation and/or bone resorption [3]. Mature osteocytes secrete sclerostin*,* a protein encoded by the *SOST* gene [4]. Sclerostin is a potent antagonist of Wnt signaling. It inhibits bone resorption by regulating osteoclastogenesis and promotes bone formation by regulating osteoblastogenesis [4–6]. Mechanical loading downregulates *SOST* expression, while disuse or a lack of loading causes an upregulation both in vivo and in vitro [7, 8]. Osteocytes induce osteoclastogenesis by increasing receptor activator of nuclear factor kappa-B ligand (RANKL) expression and by decreasing osteoprotegerin (OPG) expression, or conversely, the proportions can be reversed to inhibit bone resorption [9]. Moreover, osteocytes also function as endocrine cells to regulate phosphate homeostasis in the kidney by producing fibroblast growth factor 23 (FGF23) [9, 10]. FGF23 decreases 1,25-dihydroxyvitamin D_3_ (1,25(OH)_2_D_3_) and parathyroid hormone (PTH) concentrations to maintain blood phosphate and calcium levels, while the synthesis and secretion of FGF23 itself is regulated by 1,25(OH)_2_D_3_ and PTH [11, 12]. Thus, osteocytes play a crucial role in bone homeostasis, as well as in the regulation of blood mineral and hormone levels. However, the mechanism of osteocyte regulation of bone remodeling in response to mechanical loading and phosphate homeostasis is still not fully understood.

Most studies use isolated primary osteocytes or osteocyte-like cell lines such as MLO-Y4 cells in monolayer culture [13–16]. However, these models lack the 3D cell distribution that is highly important in determining the mechanoresponsiveness of osteocytes. OCY454 osteocyte-like cells cultured in 3D on a porous polystyrene scaffold exhibit increased *SOST* gene expression compared to monolayer culture [8]. A 3D model of human primary osteocytes seeded on biphasic calcium phosphate microbeads demonstrated that osteocytes in 3D culture express significantly more *SOST* and *FGF23* than in 2D monolayer culture [5]. Human primary osteocytes in these 3D microbeads, but not in 2D monolayer, respond to PTH with decreased *SOST* gene expression and increased *RANKL* relative to *OPG* gene expression [6]. These findings indicate that osteocytes in 3D better replicate the osteocyte phenotype in vivo and are therefore superior to 2D-cultured osteocytes [5, 6, 8]. It remains a challenge to isolate a sufficient number of primary osteocytes for cell culture experiments without losing the specific osteocyte characteristics, e.g., their mechanosensing capacity.

Osteocyte activity is affected by the calcified collagenous matrix in the native environment. Elevated extracellular calcium induces a rise of cytosolic calcium in chick osteocytes, affecting their activity [17]. Moreover, failure of type I collagen cleavage by collagenase results in osteocyte apoptosis [18]. Osteocyte activity is also affected by the oxygen concentration in the native matrix of osteocytes [19]. The prevailing oxygen concentration differs between osteocytes in cortical bone (4.2%) and in monolayer culture (18.6%) [20, 21]. Hypoxia (1% O_2_) enhances mineralization and sclerostin protein expression of human primary osteocytes compared to normoxia (20% O_2_), showing that hypoxia retains the osteocyte phenotype ex vivo [19]. Hormones, such as 1,25(OH)_2_D_3_, also affect osteocyte activity [22–25]. 1,25(OH)_2_D_3_ induces *CYP24A1* [22] and *FGF23* expression [23]. Increased *CYP24A1* and *FGF23* expression result in decreased 1,25(OH)_2_D_3_ in a feedback loop to prevent excess vitamin D pathway activation [24, 25]. The native matrix of osteocytes contains cytokines such as interleukin-1β, tumor necrosis factor-α, and interleukin-6, that potentially affect osteocyte mechanoresponsiveness and signaling toward osteoclasts and/or osteoblasts [26–28]. Thus, the osteocyte microenvironment is important for their function and should be taken into account in studies on osteocyte mechanoresponsiveness. This also suggests that a model of osteocytes cultured in their native matrix is superior to a model of osteocytes cultured in 2D to mimic the in vivo osteocyte phenotype. Earlier we have shown that osteocytes cultured in their native matrix express sclerostin, and survive up to 7 days, thereby providing an in vitro platform to study osteocytes in their native matrix [29]. More knowledge regarding the effect of a changed matrix composition, as in metabolic bone disease, on osteocyte function might facilitate the development of new treatments for these diseases.

In this study, we cultured small human cortical bone explants obtained from iliac crest and fibula to study osteocytes in their native matrix. Mechanical loading was applied on bone explants using a custom-made loading apparatus [30]. In this 3D model, the osteocytes were maintained in their original environment, i.e., with their cell bodies in lacunae and their cell processes running through canaliculi, thereby connecting to other cells. Our aim was three-fold: 1) To validate whether the custom-made loading apparatus could reliably apply different magnitudes of loading on human cortical bone, 2) to study the viability and sclerostin expression of mechanically loaded human osteocytes, and 3) to investigate the effect of different loading magnitudes on bone microdamage. This model provides a 3D structure and a native microenvironment for osteocytes. It would be relevant to test whether a changed bone matrix, as in metabolic bone disease, affects osteocyte mechanosensitivity and/or mechanoresponsiveness if our model appears suitable for studying osteocyte mechanoresponsiveness in healthy bone matrix.

## Materials and Methods

### Human Cortical Bone Culture

Bone explants were collected from 13 donors (age: 21–86). Donors presented no medical history of skeletal pathology or trauma. All bone explants were obtained with donor’s consent, and all protocols were approved by the local Medical Ethical Committee of the Amsterdam University Medical Centers (2016.105). Bone from the iliac crest was collected during maxillary sinus floor elevation surgery, and fibular bone was collected during mandible reconstruction surgery. Cortical iliac crest and fibular bone explants were washed in Hanks' balanced salt solution (HBSS; Thermo Fisher Scientific, Waltham, MA, USA), incubated in 2 mg/ml type II collagenase (Worthington Biochemical, Lakewood, CA, USA) in minimal essential medium (MEM; Thermo Fisher Scientific) for 2 h in a shaking waterbath at 37 °C. Cortical bone explants were washed again twice with HBSS, and soft tissue and trabecular bone were removed. Cleaned cortical bone was cut into small explants measuring 8.0 × 3.0 × 1.5 mm (l × w × h) using a diamond disc H-345–220 (Horico, Berlin, Germany), a handpiece (KaVo, Biberach an der Riss, Germany), and a foot control (KaVo). Explants were cooled in ice-cold HBSS during cutting. They were pre-cultured for 1 or 2 days in 6-well plates (Merck KGaA, Darmstadt, Germany), containing MEM supplemented with 5% fetal bovine serum (FBS; Lonza BioWhittaker, Basel, Switzerland), 5% bovine calf serum (BCS; Thermo Fisher Scientific), 1% penicillin–streptomycin (10,000 U/mL; Thermo Fisher Scientific), and 0.5% amphotericin B solution (Merck KGaA) at 37 °C.

### Mechanical Loading

Explants were mechanically loaded by three-point bending with a custom-made loading apparatus, which generated sinusoidal displacement using a computer-driven voice coil linear microactuator (type NCM04-25-250-2LVE; H2W Technologies, Valencia, CA, USA) as described earlier [30–32]. Explants were placed in a chamber (6.0 × 5.5 × 5 cm; l × w × h) supplemented with MEM containing 5% FBS, 5% BCS, 1% penicillin–streptomycin, and 0.5% amphotericin B solution. The two ends of an explant were placed on a custom-made bone holder. The indenter for displacement application was positioned in the center of the explant. The force exerted on the explant and the displacement were monitored and registered simultaneously [31]. The force and displacement data were recorded by a custom-made algorithm (implemented in LabVIEW 8.2, National Instruments, Austin, TX, USA) [30]. Application of mechanical loading resulted in defined bone deformation or microstrain (µɛ). The displacement of the micro-actuator was calculated by the following formula [33]:

$${\text{Displacement}} = \left( {{{12 \times {\raise0.7ex\hbox{$1$} \!\mathord{\left/ {\vphantom {1 2}}\right.\kern-\nulldelimiterspace} \!\lower0.7ex\hbox{$2$}}{\text{explant}}\;{\text{thickness}} \times {\text{strain}}} \mathord{\left/ {\vphantom {{12 \times {\raise0.7ex\hbox{$1$} \!\mathord{\left/ {\vphantom {1 2}}\right.\kern-\nulldelimiterspace} \!\lower0.7ex\hbox{$2$}}{\text{explant}}\;{\text{thickness}} \times {\text{strain}}} {\left( {{\text{loading}}\;{\text{length}}} \right)^{2} }}} \right. \kern-\nulldelimiterspace} {\left( {{\text{loading}}\;{\text{length}}} \right)^{2} }}} \right)$$

where the loading length is the length of the loaded part of the explant, which was calculated as the distance between two holding points of the explant. The thickness of the explant was calculated as the mean of the thickness of the two ends and the thickness at the center of the explant using a calibrator (Nakamura Mfg, Tokyo, Japan). Different displacements were applied on explants representing different loading magnitudes (range: 2302–13,811 µɛ) to assess the relationship between loading magnitude and force. To ensure contact between explant and indenter, a pre-load of 0.5 or 1 N was applied prior to mechanical loading to ensure contact between explant and indenter [30]. Thereafter, explants were subjected to a 5 min bending at different magnitudes (range: 2302–13,811 µɛ) and at different frequencies (1, 1.5, 2, and 5 Hz). To determine a suitable regime to keep the contact between explant and indenter, two pre-loads (0.5 and 1 N) and four different loading frequencies (1, 1.5, 2, and 5 Hz) were tested. Contact preservation at different frequencies and different pre-load magnitudes was determined by calculating the percentage of the force at the end of the mechanical loading to the initial pre-load force applied. The displacement preservation at different frequencies was expressed as the percentage of the displacement at half duration of the mechanical loading to the initial displacement. Immediately after mechanical loading, explants were either or not post-cultured for 24 h in MEM containing 5% FBS, 5% BCS, 1% penicillin–streptomycin, and 0.5% amphotericin B solution.

### Cell Viability

Explants were collected at day -2 (pre-culture), day 0 (pre-load), day 1 (without loading at 24 h post-culture), and day 1 (with 1600 µɛ loading for 5 min at 1 Hz at 24 h post-culture). Explants were incubated with freshly prepared lactate dehydrogenase (LDH) solution containing 5% polypep (Merck KGaA), 2 mM Gly-Gly (Merck KGaA), 0.75% NaCl (Thermo Fisher Scientific), 60 mM lactic acid (Merck KGaA), 1.75 mg/mL ß-nicotinamide adenine (Merck KGaA), and 30 mg nitro blue tetrazolium (Merck KGaA), pH 8.0, for 24 h in a shaking water bath at 37 °C. Then, explants were washed twice with phosphate-buffered saline (PBS) and fixated in 4% paraformaldehyde for 24 h at 4 °C [34]. Fixated explants were washed twice with PBS, dehydrated in graded ethanol series, and embedded in methylmethacrylate (MMA; BDH Chemicals, Poole, UK). Five µm thick sections were cut using a Polycut S (SM2500) microtome (Leica/Reichert-Jung, Nussloch, Germany). Sections were mounted in depex (Avantor, Radnor, PA, USA) and covered by cover slips. Digital images of LDH-stained explants were captured with a microscope (Eclipse 80i; Nikon, Tokyo, Japan), equipped with a DS-Fi1 camera (Nikon) and NIS-Elements AR software (version 4.13, Nikon). For each section, one focused image was generated from 15 to 31 images captured at 3 or 5 µm Z-intervals with a 20 × /0.5 NA objective (CFI Plan Fluor, Nikon). Blue LDH-stained viable osteocytes and empty osteocyte lacunae were counted at × 200 magnification. The percentage of viable osteocytes was calculated in relation to the total number of osteocytes (i.e., total number of empty and osteocyte-filled lacunae).

### Sclerostin Immunostaining

Explants collected at day -1/-2 (pre-culture), day 0 (preloading), day 1 (without loading at 24 h post-culture), and day 1 (loaded by 2000 or 8000 µɛ for 5 min at 1 Hz, at 24 h post-culture) were embedded in MMA and cut into 5 µm thick sections as described under “cell viability.” These sections were rehydrated, and endogenous peroxidase was quenched with 3% H_2_O_2_ in a mixture of 40% methanol in PBS. Antigen retrieval was performed by incubation with proteinase K for 15 min at 37 °C. The sections were incubated with 1/100 mouse-anti-sclerostin antibody, RRID:AB_2195349 (Bio-Techne, Minneapolis, MN, USA) for 3 h at room temperature, followed by incubation with Envision mouse-specific antibody (Agilent, Santa Clara, CA, USA) for 30 min. For color development, sections were incubated with DAB-Nickel substrate and counterstained with Nuclear fast red. Sclerostin staining was visualized using a microscope (Eclipse 80i; Nikon), equipped with a DS-Fi1 camera (Nikon) and NIS-Elements AR software (version 4.13, Nikon). For each section, one focused image was generated from 19 to 25 images captured at 3 µm or 5 µm Z-intervals with a 20 × /0.5 NA objective (CFI Plan Fluor, Nikon). The number of sclerostin-positive osteocytes and the total number of osteocyte lacunae were counted in three areas of interest of the same size (2.94 or 2.16 mm^2^) in each section: two areas of interest were located at the two ends of the section, and one area of interest was located in the center of the section.

### Real-time PCR

Cortical explants cultured for 7 days were treated without or with 10^–7^ M 1,25(OH)_2_D_3_ for 24 h. TRIzol® Reagent (Thermo Fisher Scientific) was added for 30 min. Total RNA was isolated from osteocytes according to the manufacturer’s protocol (Thermo Fischer Scientific). The RNA concentration was determined using a Nanodrop spectrophotometer (Nanodrop Technologies, Wilmington, DE, USA). RNA was reverse transcribed from 100 ng total RNA in a 20 µL reaction mixture as described [35]. The PCR reaction was performed on 25 µL reaction mixture containing 3 µL cDNA, 300 nmol/L forward and reverse primer and iQ™ SYBR Green Supermix (Bio-Rad, Hercules, CA, USA). The following primer sets were used: *CYP24A1*, forward: 5′-CAAACCGTGAAGGCCTATC-3′ reverse: 5′-AGTCTTCCCCTTCCAGGATCA-3′; *FGF23*, forward: 5′-TGAGCGTCCTCAGAGCCTAT-3′, reverse: 5′-TTGTGGATCTGCAGGTGGTA-3′; *GALNT3,* forward: 5′-AGGAACGTGGGGAAGCTAAA-3′, reverse: 5′-GTCGAGTGTCTGGTCCAAGA-3′; *PHEX*, forward: 5′-CCGAAGCCATACAGAAAGCCT-3′, reverse: 5′-CGGAAAGGTGAATGCCGTAG-3′; *RANKL,* forward: 5′-CGGGGTGACCTTATGAGAAA-3′, reverse: 5′-GCGCTAGATGACACCCTCTC-3′; *SOST*, forward: 5′-ACCACCCCTTTGAGACCAAAG-3′, reverse: 5′-GGTCACGTAGCGGGTGAAGT-3′. PCR was performed on an iCycler iQ™ RealTime PCR Detection System (Bio-Rad): 3 min at 95 °C, 40 cycles consisting of 15 s at 95 °C and 1 min at 60 °C. Relative gene expression was calculated by the 2^∆Ct^ method. TATA binding protein (*TBP*) gene was used as a housekeeping gene (sequence, forward: 5′-AGTTCTGGGATTGTACCGCA-3′, reverse: 5′-TCCTCATGATTACCGCAGCA-3′).

### Micro-computed Tomography Analysis

Explants were cut as described under “mechanical loading” and kept in 12-wells plates (Corning, New York, NY, USA) in water at 4 °C. Explants were scanned by micro-CT (Scanco Medical A.G., model μct40, Basel, Switzerland). The tube voltage of the micro-CT scanner was 70 kVp, and the tube current of 0.114 mA. The scanning resolution was 18 micron. Explant images were reconstructed by measuring the radiolucency of the object with sensors and rotating X-ray beams from different angles and subsequent calculation of the 3D structure. Bone volume (BV) was determined using the standard method of the micro-CT evaluation program [36]. The threshold was 560 mg hydroxyapatite (HA)/cm^3^, indicating that every voxel with a bone density value above 560 mg HA/cm^3^ was defined as bone.

### Quantification of Microdamage

Barium sulfate (BaSO_4_) staining was used as a contrast enhancer for microdamage detection in explants as described [37]. BaSO_4_ precipitates in microcracks, resulting in a high density on micro-CT scans. The explants were soaked in a solution of equal parts 0.9% NaCl, acetone (Avantor), and 0.5 M BaCl_2_ (Merck KGaA) in demineralized water for 3 days. Then, the explants were washed in demineralized water to remove excess ions and particles from the explant surface. Then, the explants were soaked in a solution of equal parts 0.9% NaCl, acetone, 0.5 M Na_2_SO_4_ (Merck KGaA) in demineralized water for 3 days, washed with demineralized water, and scanned by micro-CT. Explants were kept under vacuum (900 mBar) during the 6 days of preparation. All solutions were neutralized to pH 7.0 before use.

Explants were scanned 3 times, i.e., before loading, after loading (500, 1500, 2500, 4500, 6500, and 7500 µɛ at 1 Hz with a pre-load of 0.95–1.05 N for 5 min), and after loading without/with BaSO_4_ staining. Explants were scanned perpendicular to the longitudinal axis. The bone mineral density (BMD) of 3 scans was used to quantify the microdamage caused by loading. The micro-CT scans of unloaded, loaded, and BaSO_4_-stained loaded explants were transformed to the same position using a custom-made 3D rigid registration program (Academic Center for Dentistry Amsterdam (ACTA), Amsterdam, The Netherlands). The micro-CT scans of unloaded explants were used as the destination position for the micro-CT scans of mechanically loaded BaSO_4_-stained explants. A voxel mask was produced consisting of all overlapping voxels in the 3 micro-CT scans that had been rotated to the same position. To prepare a BMD graph, a mask was applied to the 3 micro-CT scans to remove ill-defined staining values. Five surface layers were deleted to remove precipitated BaSO_4_ on the free surface of the explants, which did not represent microcracks. The BMD of the unloaded micro-CT scan was subtracted from that of the mechanically loaded BaSO_4_-stained micro-CT scan, which was used to calculate the difference in BMD between the two scans. The difference in BMD was at the most 300 native units. Therefore, all voxels with a value above 300 native units were considered as BaSO_4_-stained volume (SV). The ratio of BaSO_4_-stained volume and bone volume (SV/BV) was determined as microdamage in explants. 3D images of BaSO_4_-stained volume in explants were made with para-view program (para-view version 5.6, Kitware Inc., New York, NY, USA).

### Statistical Analysis

**Fig. 1:**
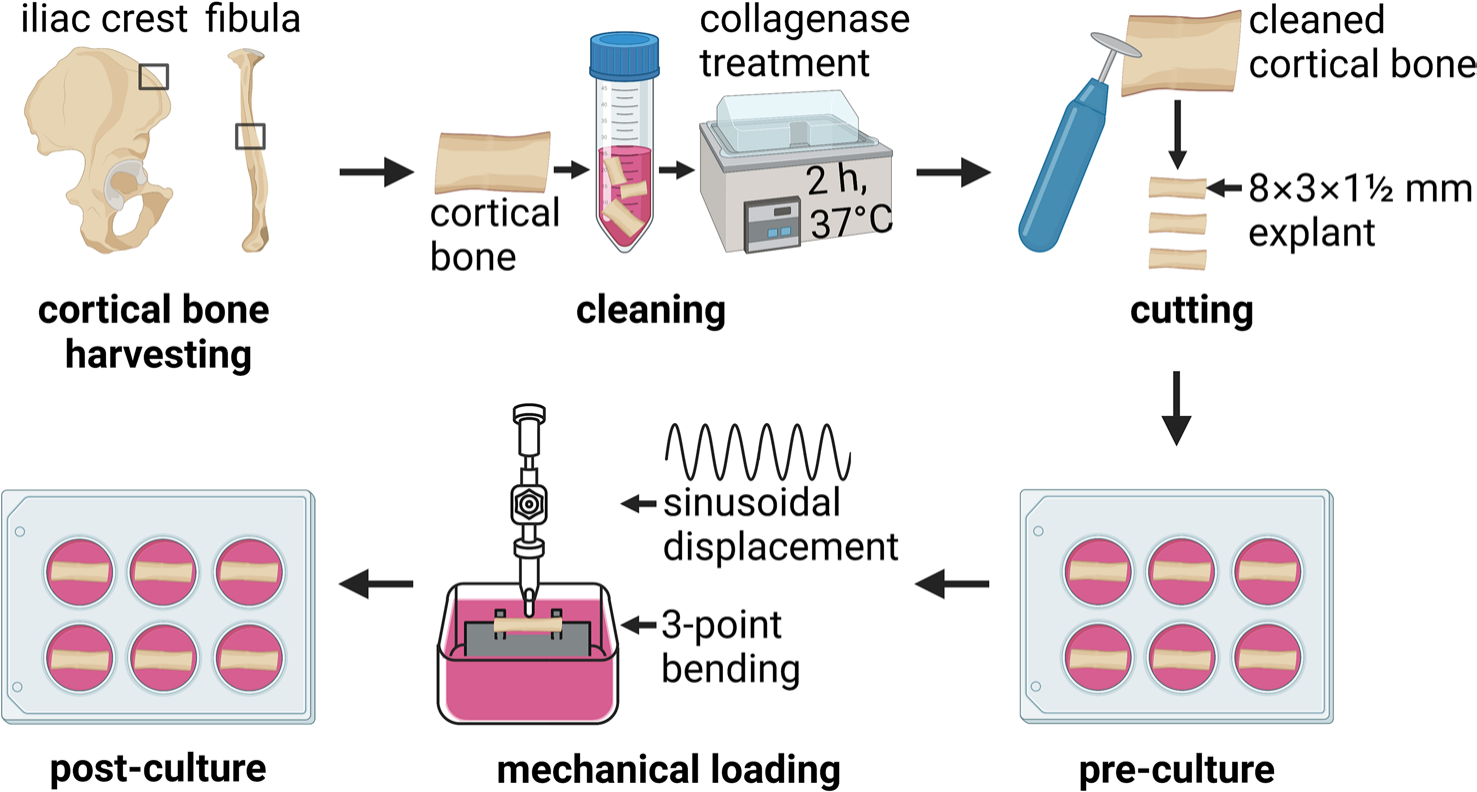
Schematic overview of human cortical bone explants preparation, mechanical loading using the custom-made loading apparatus, and culture. Cortical iliac crest bone was harvested during maxillary sinus floor elevation surgery, and fibular bone was harvested during mandible reconstruction surgery (indicated by squares). Cortical iliac crest bone and fibular bone were incubated in 2 mg/ml type II collagenase in MEM for 2 h in a shaking waterbath at 37 °C. Cleaned cortical bone was cut into small explants measuring 8.0 × 3.0 × 1.5 mm (l × w × h) using a diamond disc and a handpiece. Explants were pre-cultured in 6-well plates with MEM supplemented with 5% FBS, 5% BCS, 1% penicillin–streptomycin, and 0.5% amphotericin B solution at 37 °C. Explants were mechanically loaded for 5 min by three-point bending with a custom-made loading apparatus, which generated sinusoidal displacement. During loading, explants were placed in a chamber (6.0 × 5.5 × 5 cm; l × w × h) supplemented with MEM plus additives. The two ends of an explant were placed on a custom-made bone holder. The indenter for displacement application was positioned in the center of the explant. The displacement of the micro-actuator was calculated by the formula Displacement = (12 × ½ explant thickness ×  strain/(loading length)^2^). Immediately after mechanical loading, explants were either or not post-cultured for 24 h in MEM plus additives. Figure created with BioRender.com

**Fig. 2:**
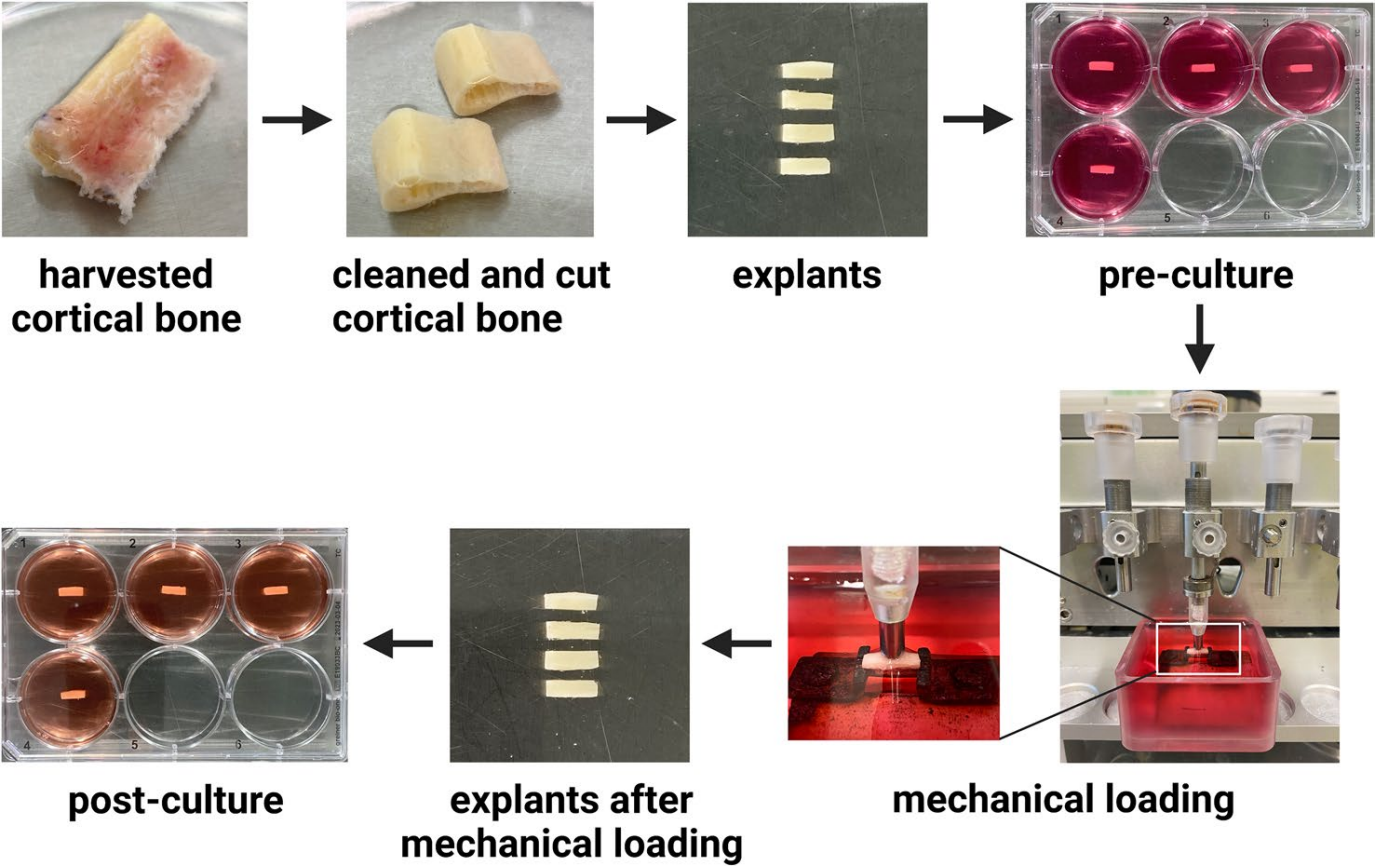
Images of human cortical bone explants during preparation, mechanical loading using the custom-made loading apparatus, and culture. Cortical iliac crest was harvested during maxillary sinus floor elevation surgery, and fibular bone was harvested during mandible reconstruction surgery. Cortical bone was cleaned and cut into small explants measuring 8.0 × 3.0 × 1.5 mm (l × w × h). After pre-culture for 1 or 2 days, explants were mechanically loaded for 5 min. Immediately thereafter, mechanically loaded explants were either or not post-cultured for 24 h. Figure created with BioRender.com

Linear regression analysis was used to determine the relationship between displacement and force, as well as between microstrain and force. Unpaired t-test was used to compare the contact preservation during loading with different pre-loads and the displacement preservation during loading at different frequencies. One-way ANOVA test and unpaired t-test were used to compare the percentage of sclerostin-positive osteocytes in different groups. Paired *t*-test was used to compare differences in gene expression between control and 1,25(OH)_2_D_3_-treated bone. One-way ANOVA and unpaired *t*-test were used to compare differences in SV/BV between explants loaded at different magnitudes. All analyses were performed using GraphPad Prism software 9 (GraphPad, San Diego, CA, USA). A p-value < 0.05 was considered significant (Figs. 1, 2).

## Results

### Mechanical Loading of Human Cortical Bone Using a Custom-Made Loading Apparatus

**Fig. 3:**
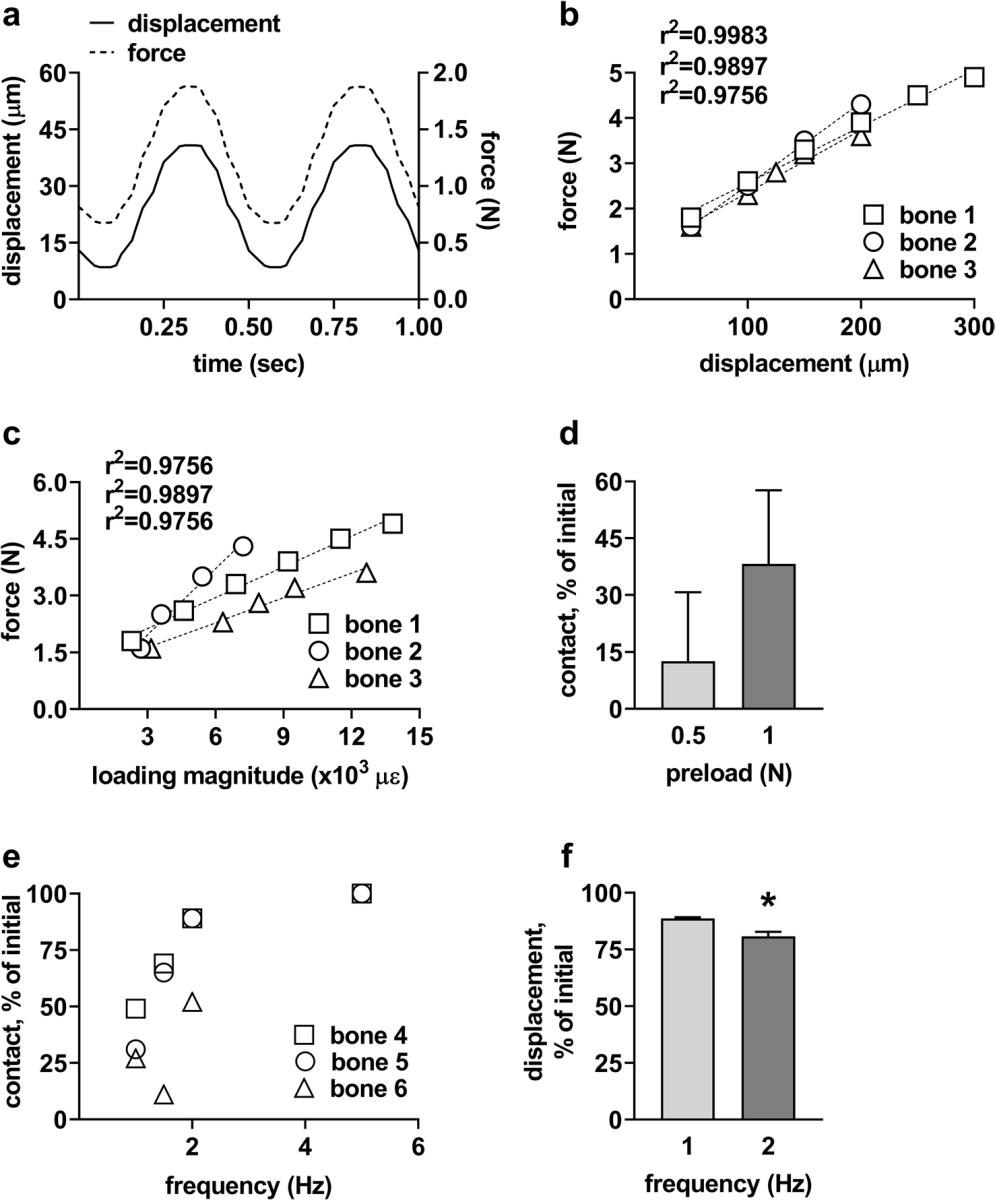
Application of defined mechanical loading on human cortical bone explants using a custom-made loading apparatus. **a** Sinusoidal displacement of indenter and force exerted on explants were monitored and registered simultaneously. **b** Dose–response relationship between indenter displacement and force on 3 separate explants (bone 1, 2, 3) obtained from 2 different patients. **c** Dose–response relationship between loading magnitude and force on 3 separate explants (bone 1, 2, 3) obtained from 2 different patients. **d** Contact preservation between explant and indenter with different pre-load. Contact preservation was expressed as percentage of force at the end of loading to the initial pre-load applied (*n* = 6). **e** Contact preservation between explant and indenter at different mechanical loading frequencies (*n* = 3). **f** Displacement preservation at mechanical loading frequencies of 1 and 2 Hz. Displacement preservation was expressed as percentage of displacement at half duration of loading to the initial displacement (*n* = 7). Values are mean ± SD. *Significantly different from 1 Hz, *p* < 0.01

To determine whether it is possible to reliably apply a defined magnitude of loading on human cortical bone explants by three-point bending using the custom-made loading apparatus, we assessed the relationship between the applied displacement or magnitude and force. Sinusoidal displacement and force on explants were recorded simultaneously during loading (Fig. 3a). There was a significant positive linear relation between the displacement or microstrain and the force in 3 explants (*p* < 0.05; Fig. 3b, c). When the explants were loaded by the same displacement (50, 100, 150, 200 µm), the force exerted on the 3 explants was similar, demonstrating that the custom-made loading apparatus did reliably apply mechanical loading on different explants (Fig. 3b).

Contact preservation between the explant and the indenter during loading is essential for reliably applying mechanical loading on the explant. Pre-loads and frequencies of different magnitudes were applied to investigate whether this would affect the contact between explant and indenter during loading. A pre-load of 1 N followed by loading slightly, but not significantly, improved contact preservation compared to a pre-load of 0.5 N (Fig. 3d). Therefore, a pre-load of 1 N was chosen for further experimentation. When explants were loaded at a frequency of 1, 1.5, 2, or 5 Hz, the contact between explant and indenter was preserved during loading (Fig. 3e). To test whether the loading frequency affected the displacement of the indenter, explants were loaded at 1 or 2 Hz. Loading at 1 Hz showed improved displacement preservation compared to loading at 2 Hz (*p* < 0.05; Fig. 3f). Therefore, a loading frequency of 1 Hz was chosen for further experimentation.

### Cell Viability of Mechanically Loaded Osteocytes in Their Native Matrix

**Fig. 4:**
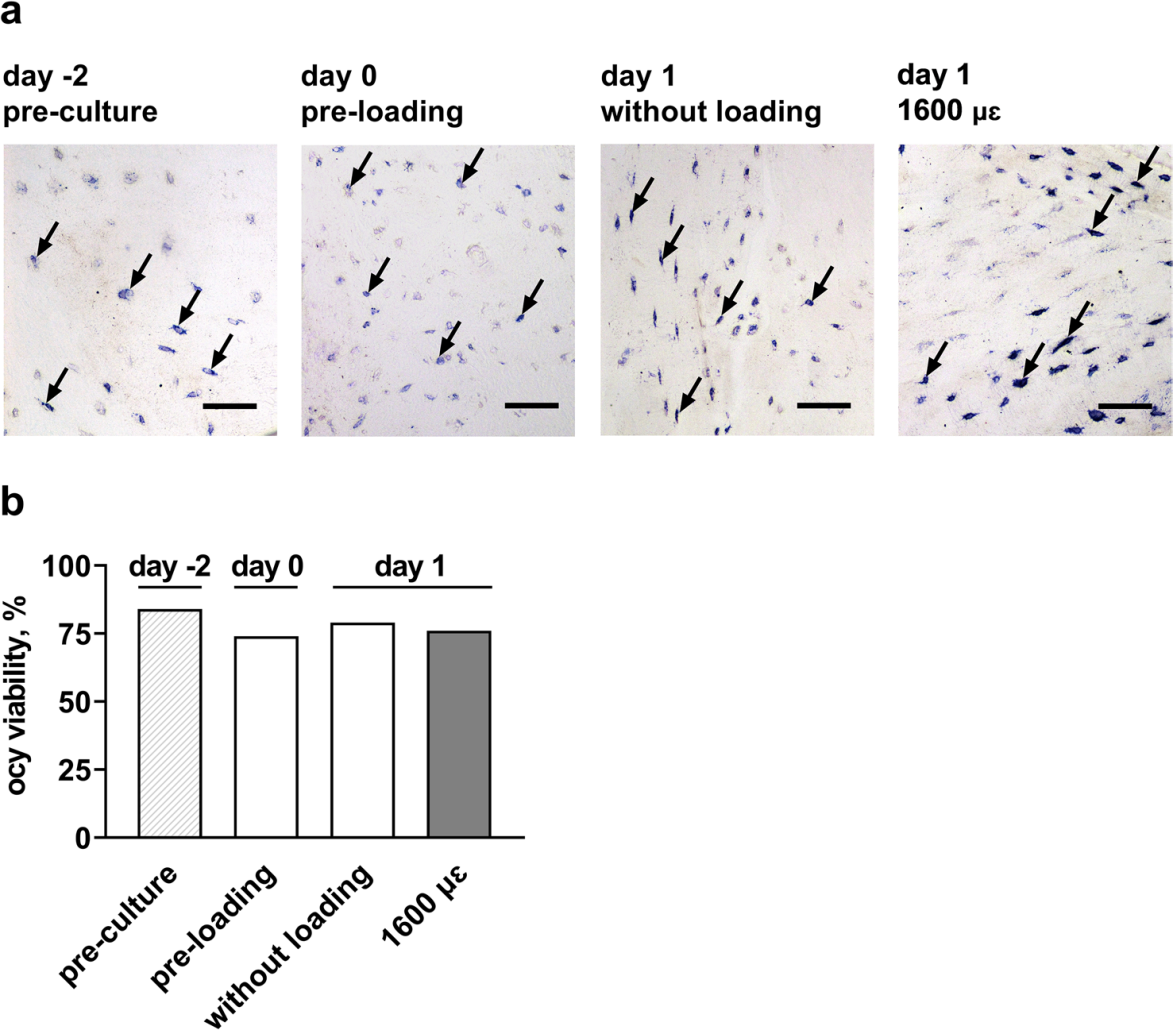
Mechanical loading did not affect osteocyte viability 24 h after loading**. a** Microphotographs showing LDH-positive osteocytes (blue) in human cortical bone explants. Black arrows: LDH-positive osteocytes in their lacunae; scale bar: 50 µm. **b** Quantification of osteocyte viability. Values are mean percentage of LDH-positive osteocytes to total osteocytes, in explants from 2 donors. Ocy, osteocytes; LDH, lactate dehydrogenase

The viability of osteocytes before and after mechanical loading was tested (Fig. 4a). The percentage of viable osteocytes was similar in all groups, i.e., 84% in pre-culture explants (day -2), 74% in pre-loading explants (day 0), 79% in explants without loading (day 1), and 76% in explants with 1600 µɛ loading at 24 h post-culture (day 1; Fig. 4b). Thus, loading at 1600 µɛ did not affect osteocyte viability at 24 h post-culture.

### Sclerostin Expression by Mechanically Loaded Osteocytes in Their Native Matrix

**Fig. 5:**
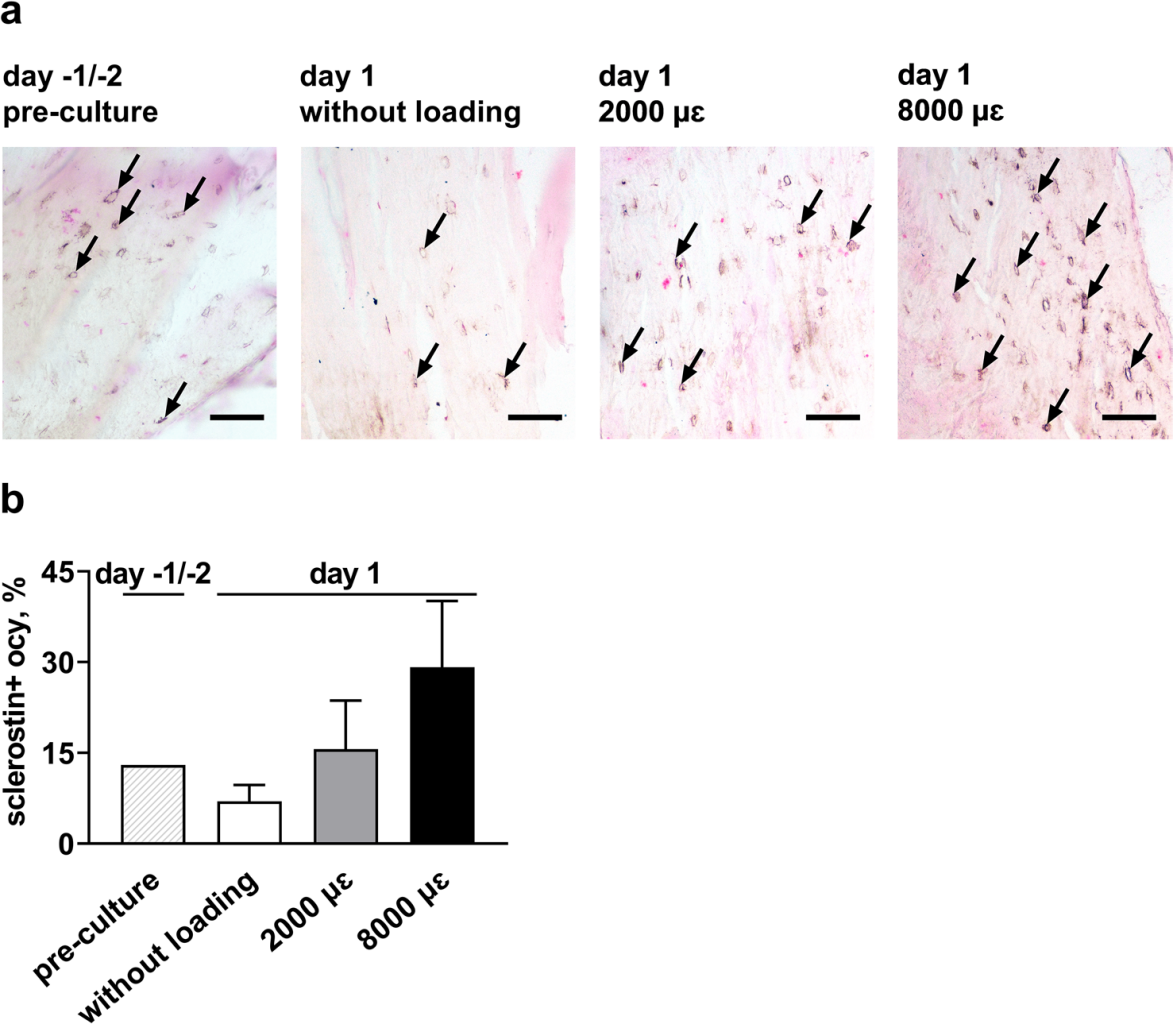
Sclerostin expression in osteocytes slightly increased with increasing mechanical loading magnitude up to 8000 µɛ. **a** Microphotographs of immune-histochemical detection of sclerostin in human cortical bone explants. Explants were analyzed at day -1/-2 prior to loading at day 0. At day 1, explants without loading, and explants loaded by 2000 or 8000 µɛ, were analyzed. Arrows: sclerostin-positive osteocytes; scale bar: 50 µm. **b** Percentage of sclerostin-positive osteocytes to total osteocytes. There was no significance between groups, p > 0.05. Values are mean ± SD of data from 1 donor. Sclerostin + ocy, sclerostin-positive osteocytes

The phenotypic stability of mechanically loaded osteocytes was investigated by measuring sclerostin protein expression. The number of sclerostin-positive stained osteocytes and the total number of osteocytes were counted at 24 h post-culture (Fig. 5a). The percentage of sclerostin-positive osteocytes was similar in all groups, i.e., 13% in pre-culture explants (day -1/-2), 7% in explants without loading (day 1), 16% in 2000 µɛ loaded explants, and 29% in 8000 µɛ loaded explants (day 1; Fig. 5d).

### 1,25(OH)_2_D_3_ Stimulation of Osteocytes in Their Native Matrix

**Fig. 6:**
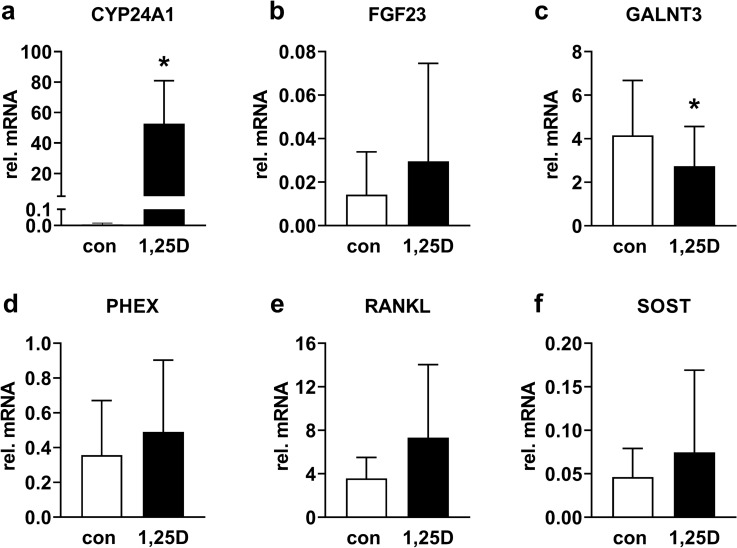
One day 1,25(OH)_2_D_3_ (10^–7^ M) treatment increased *CYP24A1* expression, decreased *GALNT3* expression, but did not affect *FGF23, PHEX, RANKL,* and *SOST* gene expression in osteocytes in their native matrix of human cortical bone after 1 week of culture. Osteocytic gene expression of: **a** *CYP24A1*, **b** *FGF23*, **c** *GALNT3*, **d** *PHEX*, **e** *RANKL*, **f** *SOST*. Values are mean ± SD of data from 5 donors. *Significant effect of 1,25(OH)_2_D_3_, p < 0.05. Con, control; 1,25D, 1,25(OH)_2_D_3_

To study whether osteocytes in our mechanical loading model keep their phenotype and respond to 1,25(OH)_2_D_3_, we tested the effect of 10^–7^ M 1,25(OH)_2_D_3_ on *CYP24A1, FGF23, GALNT3, PHEX, RANKL, and SOST* gene expression. 1,25(OH)_2_D_3_ treatment significantly increased *CYP24A1* expression (*p* < 0.05, Fig. 6a), indicating that osteocytes sensed and responded to 10^–7^ M 1,25(OH)_2_D_3_. 1,25(OH)_2_D_3_ treatment did not affect *FGF23* gene expression (Fig. 6b), but significantly decreased *GALNT3* gene expression (*p* < 0.05, Fig. 6c). *PHEX*, *RANKL*, and *SOST* gene expression were not affected by 1,25(OH)_2_D_3_ (Fig. 6d–f). The fact that osteocytes expressed osteocyte-specific markers *FGF23 and SOST* demonstrated that the osteocytes maintained their osteocytic phenotype in our mechanical loading model.

### Mechanical Loading at Different Magnitudes and Explant Microdamage

**Fig. 7:**
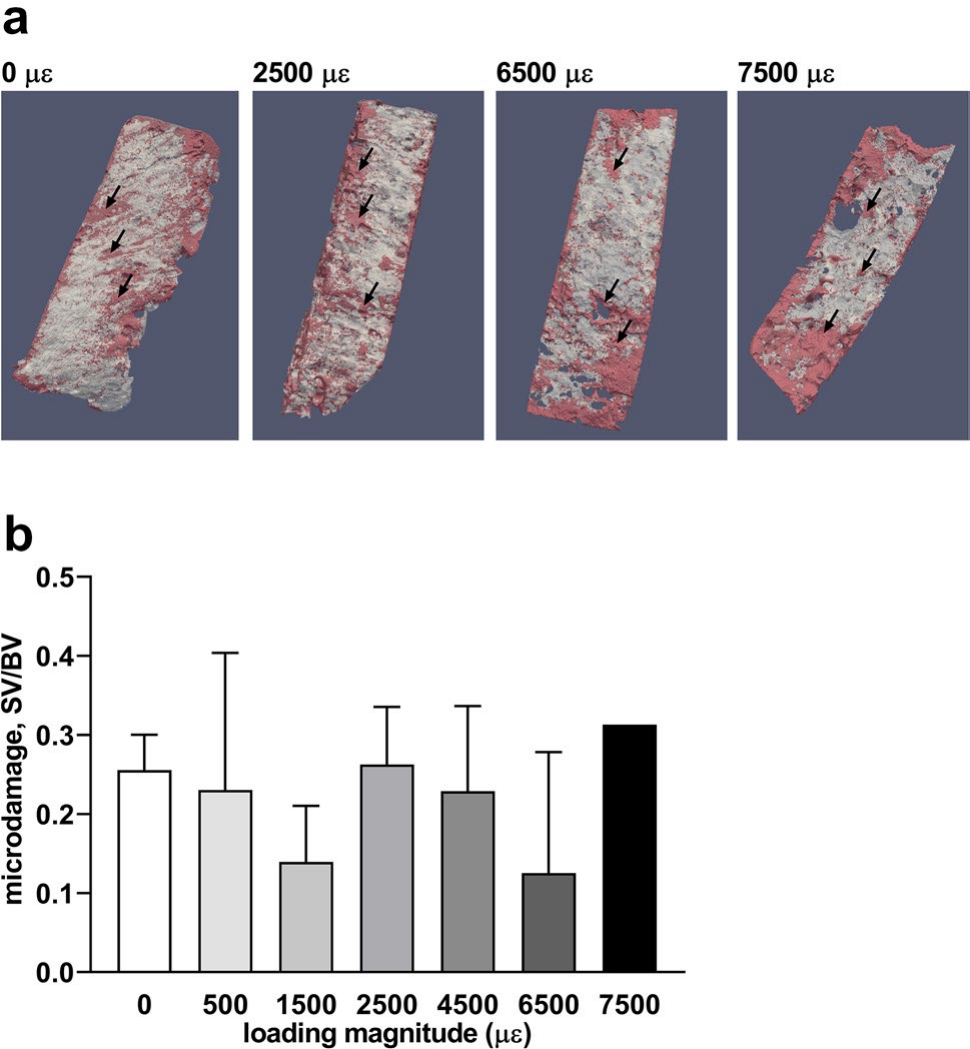
Mechanical loading up to 7500 µɛ did not increase microdamage in bone. **a** 3D images of unloaded explants (0 µɛ), and explants loaded at 2500, 6500, and 7500 µɛ at 1 Hz for 5 min. Red area: BaSO_4_-stained microdamage (black arrows). Gray area: not-damaged bone. **b** Microdamage determined as BaSO_4_-stained volume/bone volume (SV/BV) of unloaded (0 µɛ) and loaded explants (500, 1500, 2500, 4500, 6500, 7500 µɛ). There was no significant difference between different loading magnitudes. Values are mean ± SD of data from 3 donors

To investigate whether loading at different magnitudes causes explant microdamage, BaSO4 staining was performed to indicate and quantify microdamage. Explants were either unloaded or loaded at 500 µɛ to 7500 µɛ. 3D images of mechanically loaded explants revealed the location of BaSO_4_-stained bone indicating microdamage (Fig. 7a). The amount of microdamage was similar in unloaded and loaded explants (Fig. 7b). This indicated that mechanical loading up to 7500 µɛ did not increase microdamage compared to unloaded explants.

## Discussion

In this study, a 3D mechanical loading model of human osteocytes in their native matrix was developed and validated. Using our custom-made loading apparatus, a dose–response relationship between displacement and force and between magnitude and force was observed. The osteocytes in bone exposed to mechanical loading of 1600–8000 µɛ were highly viable at 24 h post-culture. The osteocytes in this model maintained their osteocytic phenotype, as shown by sclerostin expression, as well as by gene expression of osteocyte-specific markers *FGF23* and *SOST*. Mechanical loading up to 7500 µɛ did not increase explant microdamage. These findings indicate that our 3D mechanical loading model is suitable for investigating the mechanosensitivity and mechanoresponsiveness of human osteocytes in their native matrix.

Our 3D mechanical loading model used a custom-made loading apparatus to apply a well-defined magnitude of mechanical loading on human cortical bone. The observed linear relationship between the loading magnitude (2302–13,811 µɛ) and force on explants from two donors indicated that this model allows application of different loading magnitudes on explants from different donors*.* This agrees with published data also showing a dose–response relationship between force and microstrain in mouse ulna bone [38]. Moreover, we compared the effect of pre-load (0.5 or 1 N) on contact preservation since preliminary data showed that the contact between explant and indenter was not preserved during the loading session, due possibly to a change in bone shape during loading. If the contact is not preserved, the displacement of the indenter applied on the explant will decrease, resulting in unreliable strain calculations. We found that 1 N pre-load followed by mechanical loading promoted contact preservation compared to 0.5 N pre-load. Moreover, it has been reported that mechanical loading at 1 N generates little bone formation [38]. Therefore, to maintain contact between explant and indenter, and to minimize the effect of pre-load on osteocyte mechanoresponsiveness, we choose for a 1 N pre-load. We also tested whether the contact between explant and indenter was affected by frequency. All contact was preserved between explant and indenter at frequencies of 1, 1.5, 2, and 5 Hz.

The frequency of mechanical loading did not only affect the contact preservation between explant and indenter, but also the accuracy of indenter displacement. We found that mechanical loading at 1 Hz improved displacement preservation compared to loading at 2 Hz. These frequencies were chosen based on published data showing that most strain energy in walking dogs is contained between 0 and 2 Hz [39]. Moreover, loading at 1610, 2640, 3660, and 4680 µɛ at 1 Hz induces rat ulna cortical bone formation in a dose–response manner [40]. Therefore, loading at 1 Hz frequency might trigger an osteocytic response. The frequency of mechanical loading affects bone adaption to mechanical loading, i.e., increased frequencies (1, 5, and 10 Hz) result in more robust bone formation [40], as well as increased bone mass in the equilibrium state in a mathematical trabecular bone remodeling model [41]. Similarly, cortical bone formation is enhanced with increased frequencies up to 5–10 Hz and plateaus at frequencies beyond 10 Hz [38]. In our study, with the limitation of limited displacement preservation at frequencies higher than 1 Hz, we utilized mechanical loading at a frequency of 1 Hz. Future studies are needed to investigate the effect of a combination of different loading frequencies and magnitudes on osteocyte mechanoresponsiveness.

Our 3D mechanical loading model was able to maintain high osteocyte viability in both unloaded bone (79%) and in bone at 24 h post-culture (76%), demonstrating that 5 min mechanical loading at 1600 µɛ and 1 Hz did not influence cell viability. This suggested that our model is useful for testing mechanosensitivity of osteocytes with a pre-culture time up to 2 days. Inclusion of a pre-culture period raises the possibility to incubate bone with small molecules, such as mechanoregulating proteins, that will facilitate studies on the role of specific molecules in osteocyte mechanosensing and mechanoresponsiveness. These small molecules likely reach the osteocytes in bone, as indicated by the osteocytes’ response to exogenous 1,25(OH)_2_D_3_ stimulation. Osteocytes showed an upregulation of *CYP24A1* gene expression in response to 10^–7^ M 1,25(OH)_2_D_3_ treatment. This indicated that they maintained their responsiveness to 1,25(OH)_2_D_3_, which is a small molecule that can diffuse within the calcified bone matrix to reach the embedded osteocytes. Moreover, gene expression of *FGF23* and *SOST* was observed after 7 days of culture, suggesting that viable osteocytes maintained their osteocyte phenotype. The *SOST* gene is not expressed in the osteocyte cell line MLO-Y4, and the *FGF23* gene is not expressed in isolated human primary osteocytes, but both SOST and FGF23 are expressed in 3D structured and hypoxia-conditioned osteocytes [19, 42, 43]. We also showed that osteocytes in loaded human cortical bone did secrete sclerostin. Therefore, our model appears superior over osteocyte cell lines and mimics in vivo osteocytes in native bone. Mechanosensitive gene expression is a common outcome parameter in studies on mechanosensitivity and responsiveness of osteocytes, and therefore, our results indicate the suitability of our model to study osteocyte mechanoresponsiveness. Taken together, the osteocytes in their native matrix maintained their osteocytic phenotype up to 7 days and responded to changes in their microenvironment. This suggests that our model can be used to study the effect of a changed matrix, such as in patients with bone metabolic disorders, and the effect of small molecules, such as cytokines and hormones, on the mechanoresponsiveness of human osteocyte in their native matrix.

We found that mechanically loaded osteocytes revealed more clear cellular processes and bigger cell bodies than unloaded cells. Mechanical loading might increase osteocyte activity, as well as the connection with other osteocytes and osteoblasts and/or osteoclasts. Osteocyte activity might also have decreased as a result of unloading. Unlike most studies reporting that sclerostin expression in osteocytes is downregulated by mechanical loading [4,7], we found that sclerostin expression was similar in unloaded osteocytes and in osteocytes loaded at magnitudes up to 8000 µɛ. One possible explanation for this discrepancy is that 24 h post-culture was still too early, or too late, to observe a change in sclerostin expression in osteocytes. Mechanical loading might have increased osteocyte activity and sclerostin production, while the amount of sclerostin expressed per cell as well as the total amount of sclerostin expressed was decreased. The absolute expression of sclerostin still needs to be quantified in a future study.

Mechanical loading up to 7500 µɛ did not increase microdamage accumulation. Microdamge in both unloaded and loaded explants might be caused by the surgical procedure and/or by the cutting of bone into explants. Strains of 50–100 µɛ result in disuse-induced bone remodeling, strains of 1000–1500 µɛ lead to operational microdamage, strains above 3000 µɛ induce the start of bone modeling, and strains above 25,000 µɛ cause bone fracture in young adult mammals [44]. The loading magnitudes used in the current study were below the fracture threshold, and therefore, the amount of microdamage was similar in unloaded and loaded bone. This indicated that the osteocyte response to mechanical loading was affected by the loading magnitude, but not by microdamage. Our results support the hypothesis that our in vitro loading model of osteocytes in their native matrix is a suitable model for studying osteocyte mechanosensitivity and mechanoresponsiveness in bone obtained from patients with metabolic bone diseases.

This study has some limitations. The 3D mechanical loading model cannot be used for trabecular bone since this bone is too thin, too soft, and does not have a regular shape. The bone material used was surgical waste material, which was only available in limited amounts, and cut by a handpiece. Cutting bone could cause microcracks damaging the lacunacanalicular network, disruption of communication between osteocytes and osteoblasts, and decreased osteocyte mechanoresponsiveness. The cellular network in each explant was not strictly the same. Furthermore, explants might not have been obtained from exactly the same bone location, thereby causing possible variation in the bone specifics. The osteocyte mechanoresponsiveness might also have been influenced by donor age and sex.

In conclusion, our 3D mechanical loading model of human osteocytes in their native matrix mimics the osteocyte environment in vivo and is suitable to study osteocyte mechanoresponsiveness in vitro. Whether a changed bone matrix in metabolic bone disease affects osteocyte mechanosensitivity and/or mechanoresponsiveness is still unclear, but our model seems suitable for studying the effect of changed matrix composition, as in human metabolic bone diseases, on osteocyte mechanoresponsiveness, as well as the effect of small molecules on osteocyte mechanosensitivity in drug screening.

## Data Availability

Data and meterial were stored locally at Amsterdam University Medical Centers, Vrije Universiteit Amsterdam, and are available upon request.
